# PI3K Signaling in Tissue Hyper-Proliferation: From Overgrowth Syndromes to Kidney Cysts

**DOI:** 10.3390/cancers9040030

**Published:** 2017-03-29

**Authors:** Maria Chiara De Santis, Valentina Sala, Miriam Martini, Giovanni Battista Ferrero, Emilio Hirsch

**Affiliations:** 1 Department of Molecular Biotechnology and Health Sciences, University of Torino, Torino 10126, Italy; mariachiara.desantis@unito.it (M.C.D.S.); valentina.sala@unito.it (V.S.); miriam.martini@unito.it (M.M.); 2 Department of Public Health and Pediatrics, University of Torino, Torino 10126, Italy; giovannibattista.ferrero@unito.it

**Keywords:** PI3K, mTOR, proliferation, overgrowth syndrome, polycystic kidney disease

## Abstract

The members of the PhosphoInositide-3 Kinase (PI3K) protein family are well-known regulators of proliferative signals. By the generation of lipid second messengers, they mediate the activation of AKT/PKB (AKT) and mammalian Target Of Rapamycin (mTOR) pathways. Although mutations in the PI3K/AKT/mTOR pathway are highly characterized in cancer, recent evidence indicates that alterations in the proliferative signals are major drivers of other diseases such as overgrowth disorders and polycystic kidney disease. In this review, we briefly summarize the role of the PI3K/AKT/mTOR pathway in cell proliferation by comparing the effect of alterations in PI3K enzymes in different tissues. In particular, we discuss the most recent findings on how the same pathway may lead to different biological effects, due to the convergence and cooperation of different signaling cascades.

## 1. Introduction

Tissue proliferation is a tightly regulated process in the organism, from embryonic development to adult life. Alterations in this process may cause several pathologic conditions, including cancer, overgrowth syndromes and polycystic kidney disease. One of the main regulators of cell proliferation is the PhosphoInositide-3 Kinase (PI3K)/AKT/PKB (AKT)/mammalian Target Of Rapamycin (mTOR) signaling pathway, which is a well-known target of multiple therapeutic strategies [1].

PI3Ks are a family of lipid kinases subdivided in three classes and eight isoforms. Class I PI3Ks act as dimers for p110α, p110β and p110δ, where the most common regulatory subunit is p85, while p110γ associates with the p101 and p84/p87 regulatory subunits. PI3Ks play a key role in the transduction of signals deriving from activated tyrosine kinase receptors (RTKs), G protein-coupled receptors (GPCRs), and RAt Sarcoma (RAS). The main product of class I PI3Ks is phosphatidylinositol-3,4,5-triphosphate PI(3,4,5)P3. This second messenger acts as a docking site for proteins that contain a pleckstrin homology (PH) domain, such as AKT, which is one of the major downstream effectors of PI3K and activates a series of downstream signaling pathways, including the AKT/mammalian target of rapamycin pathway (mTOR). Upon PI3K activation, AKT can be phosphorylated by mammalian target of rapamycin complex 2 (mTORC2) on serine 473 residue or by phosphoinositide-dependent kinase 1 (PDK1) on threonine 308 residue, leading to inhibition of the tuberous sclerosis protein 2 (TSC2), a well-known tumor suppressor gene. TSC1 and TSC2 form a multi-protein complex which acts as a GTPase-activating protein (GAP) for the small GTPase Rheb, thus leading to mTORC1 inhibition. AKT-dependent activation of mTORC1 results in increased protein synthesis and cell survival by direct phosphorylation of downstream effectors, such as the ribosomal S6 kinase. 

The activity of PI3K can be opposed by several phosphatases, including phosphatase and tensin homolog (PTEN), which dephosphorylates PI(3,4,5)P3 back to PI(4,5)P2 [1,2,3]. 

Differently from class I and class III, class II PI3Ks (PI3K-C2α, PI3K-C2β, PI3K-C2γ) are monomers that present distinct long N- and C-terminal domains. In contrast to class I, class II PI3Ks are predominantly involved in the regulation of vesicular trafficking and recognize PI and PI(4)P as substrates, producing PI(3)P and PI(3,4)P2, respectively [2,3,4]. A plethora of different receptors that modulate PI3K-C2α localization and activation has been reported and includes tyrosine kinase receptors, such as IR, as well as GPCR. Moreover, PI3Ks can bind small GTPases in multiple contexts. For example, PI3K-C2α is a RAB11 effector producing PI(3)P at the base of the primary cilium in the renal tissue, both during development and the adult life. The primary cilium is an organelle protruding from the cells and, in kidneys, acts as a mechanosensor of the fluid flow. Notably, the primary cilium plays a key role in the signaling pathways of several proteins, such as Polycystins 1 and 2 (PC1/2) [5], which are involved in the control of the fluid flow and tubular cell proliferation.

The deletion of PI3K-C2α in mice induced cilium elongation and signaling defects in the embryo and in kidney tubules, with a defect in Sonic Hedgehog (SH) and PC2 signaling, respectively. In the embryo, the loss of PI3K-C2α is associated with several defects, including no turning, no cardiac looping and smaller size [6]. In contrast to PI3KCA, the heterozygous loss of PI3K-C2α increased proliferation and predisposed the subject to cyst development, in genetic models of PC1/2 reduction or in response to ischemia-/reperfusion-induced renal damage. Indeed, PI3K-C2*α* modulates the trafficking of cargo proteins such as PC2 along the primary cilium, thus allowing polycystin-mediated control of proliferative signals in kidney tubular cells [7].

In this review, we report the recent findings in PI3K/AKT/mTOR pathway alterations, not only in the well-known context of cancer, but also in other proliferative disorders such as overgrowth syndrome and polycystic kidney disease.

## 2. PI3K Somatic Mutations Leading to Cancer and Proliferative Disorders

The hyper-activation of the PI3K/AKT/mTOR pathway results in significant dysregulation of cellular functions, which in turn leads to a competitive growth advantage. 

Somatic mutations and gains or losses in these genes are linked with many different solid and hematological tumors [8,9]. PIK3CA is frequently mutated in several cancer types, including breast, colorectal, endometrial, ovarian and skin tumors [10]. The frequency of mutations indicates that PIK3CA is one of the most highly mutated oncogenes in human cancers. High-throughput mutational analysis of human tumor samples revealed that PIK3CA is frequently affected by cancer-specific somatic mutations in the heterozygous state. Tumor-associated PIK3CA mutations increase the intrinsic lipid kinase activity of PI3K and provide a growth advantage together with invasive abilities [11,12]. These mutations are distributed over the gene sequence but more than 80% of them are located in three hotspots, E542 and E545 in the helical domain (exon 9) and H1047 in the kinase domain (exon 20). E542 and E545 are commonly turned into lysine whereas H1047 is frequently changed to arginine. Both E545K and H1047R can promote oncogenic transformation in vitro [13], while the latter is also able to induce tumorigenesis in mice [12,14]. According to structural and functional studies, these two hotspot mutations act in a synergistic but independent way [15,16]. Mutations of the PIK3CA gene are described in 25%–40% of breast cancers and in 30% of colorectal cancer [17], while PIK3CA mutations in endometrial cancer are often coincident with PTEN inactivation [18]. 

Interestingly, hotspot mutations occur also in noninvasive keratinocyte-derived skin lesions, including epidermal nevi and seborrheic keratosis [19,20]. In contrast, PIK3CA mutations are relatively rare in malignant melanoma (~3%), despite the critical role of PTEN inactivation in this tumor type [21].

Interestingly, in addition to their well-characterized role in cancer, postzygotic somatic mutations in PIK3CA have also recently been identified in a spectrum of overgrowth disorders including congenital lipomatous overgrowth with vascular, epidermal, and skeletal anomalies (CLOVES) syndrome, PIK3CA-related overgrowth spectrum (PROS), Proteus syndrome (PS) and other AKT-related disorders. 

Distinct nodes in the PI3K signaling pathway, from receptor proteins on the cell surface to protein kinases, are implicated in mosaic overgrowth syndromes. Notably, mutations are almost exclusively found in tissues of ectodermal and mesodermal origin, with overgrowth frequently affecting adipose, muscle and skeletal tissue [22]. These disorders are all caused by mutations affecting pathways that, by regulating cellular growth, are prominently involved in cancer. However, while some of these phenotypes predispose to malignancies, the majority do not. Generally speaking, cancer originates from a normal cell that has undergone a tumorigenic transformation as a result of genetic mutations, which is defined as the tumor-initiating cell. The cell type of origin together with genetic alterations contribute to the oncogenic phenotype, as has been recently demonstrated in breast cancer [23]. This suggests that the context (both temporal and cellular) in which the mutation occurs determines its phenotype, conferring heterogeneity to the spectrum of disease burden.

On the other hand, germline loss-of-function mutations of PTEN and TSC1/TSC2 cause PTEN hamartoma tumor syndrome and tuberous sclerosis complex. In all these conditions, the affected sites show increased activation of the PI3K and mTOR pathways, together with an increased proliferation rate of the tissue involved. 

PI3K/mTOR-dependent aberrant growth can also affect several tissues, including kidneys, where it results in cyst formation.

## 3. The PIK3CA-Related Spectrum of Overgrowth Syndromes

It has been reported that PI3K pathway components are somatically mutated in a spectrum of congenital or early-childhood-onset human disorders [24,25]. Some of these de novo mutations are usually somatic, as the corresponding germline mutations would lead to extremely pervasive developmental disorders, which would result in embryonic lethality [26]. A common feature of these disorders is the aberrant activation of cellular proliferation pathways and overgrowth, which have been therefore referred-to as “overgrowth syndromes”. Although each syndrome has a specific panel of clinical features, some overlapping characteristics can be determined. Similarly, such a phenotypic overlap was previously recognized for the large group of RAS-related syndromes, collectively referred-to as “RASopathies”, which are caused by activating mutations affecting RAS signaling components [27], and mainly occurring in the germline (albeit mosaic RASopathies have also been identified) [28].

The overgrowth syndromes comprise a wide group of clinically recognizable mutation-driven congenital malformations [29]. Among these, the PIK3CA-related overgrowth spectrum (PROS) congenital disorders are caused by a range of mosaic-activating mutations in the PIK3CA gene, coding for p110α.

The different mutations underlying PROS disorders result in moderate-to-strong constitutive activation of PIK3CA (Table 1), leading to the increased growth of the affected tissues [30]. The PROS spectrum is broad, from weak phenotypes to dramatic malformations, reflecting the effect of mutations during embryogenesis and in progenitor cells [31]. Within PROS, some distinct entities can be recognized, despite considerable overlap. 

Cutaneous defects together with developmental neurological abnormalities are normally present in macrocephaly-capillary malformation (MCAP) and CLOVES. In addition, several disorders are associated with connective tissue overgrowth, presumably arising from mutations affecting progenitor cells committed to a mesenchymal fate. Of note, MCAP can be occasionally caused by germline PIK3CA mutations [32]. Malignancies are rare in the PROS spectrum [24], even if PIK3CA is the most common mutated gene in cancer.

Another class of overgrowth diseases, the PTEN hamartoma tumor syndrome (PHTS), is caused by mutations in the PTEN tumor suppressor gene. In particular, heterozygous inactivating germline genetic lesions occur in the PTEN gene and they are responsible for 90% of Cowden syndrome (CS) cases. CS has a wide clinical spectrum, including skin abnormalities, macrocephaly and predisposition to multiple cancer types, including breast, colon and thyroid [33]. In segmental overgrowth, lipomatosis, arteriovenous malformation, and epidermal nevus (SOLAMEN), classic manifestations of CS are combined with segmental overgrowth, arteriovenous and lymphatic malformations, lipomas, and epidermal nevi. SOLAMEN symptoms are related to mosaic loss of the healthy PTEN allele in patients carrying a germline PTEN mutation [34]. This phenomenon, known as type 2 mosaicism, has been extensively documented for other genetic disorders. Interestingly, the patients do not develop malignancies in the tissues affected by PTEN loss.

In 2011, Lindhurst et al. reported a mosaic-activating AKT1 mutation that causes Proteus syndrome (PS) [35], which is associated with prominent skin and vascular malformations. In addition, the other AKT family members, AKT2 and AKT3, have also been implicated in overgrowth syndromes [32,36].

While most of the previous studies have been focused on germline mutations, thanks to deep-sequencing technologies, there is increased evidence that somatic mutations also are related to non-cancer phenotypes. However, there are no available mouse models that fully recapitulate the wide spectrum of phenotypes which characterize PI3KCA-related overgrowth syndromes.

## 4. PI3Ks Inhibitors as a Therapeutic Challenge in Overgrowth Syndromes

Due to the extensiveness of vascular malformations and tissue overgrowth, PI3K-related syndromes pose a therapeutic challenge. Sclerotherapy or surgery might improve symptoms or ameliorate patients’ appearance, but complete remission is seldom seen. As genetic alterations in PI3K/AKT/mTOR signaling have been recognized as pivotal cancer drivers, considerable efforts have been employed to generate a plethora of inhibitors for such pathway components. Several of these compounds are in clinical trials for cancer and may be reasonably re-purposed as targeted therapies for overgrowth conditions [44]. Recently, an association between somatic mutations in PIK3CA and sporadic venous malformations in mouse models have been demonstrated [45,46]. Both studies highlighted the efficacy of selective PI3Kα and mTOR inhibitors to reduce the size and proliferation of these malformations. Even if there are not any clinical trials to evaluate PI3K p110α inhibitors in PROS, pre-clinical studies in both PROS and cancers bearing PIK3CA mutations have yielded promising results [30]. 

Several AKT inhibitors are now in clinical trials for cancer. Notably, a phase I trial of ARQ 092, a pan-AKT inhibitor, is underway in children and adults with Proteus syndrome (ClinicalTrials.gov: NCT02594215). Allosteric inhibitors of mTOR, including sirolimus (rapamycin), everolimus, ridaforolimus, temsirolimus, are licensed therapies for a range of conditions [47]. Notably, off-label applications of mTOR inhibitors might be successfully exploited also in non-cancer diseases. Very recently, rapamycin has been suggested as a systemic therapy to reduce vascular malformations in patients [48,49], including those carrying PTEN mutations [50,51]. A clinical trial investigating sirolimus in PROS (ClincalTrials.gov: NCT02428296) is currently underway, with expected completion in 2017. Of greatest relevance to PI3K/AKT-related disorders are dual PI3K-mTOR inhibitors, which are currently in phase I trial for cancer [52].

Of note, patients with somatic activation of the PI3K/AKT pathway may need life-long therapy with the inhibitor. This raises concern not only for the more common adverse effects of these inhibitors, but also for unknown effects that cannot be revealed by short-term evaluation in clinical trials, this being a critical challenge which already emerged for RAS-related syndromes. Unfortunately, data from preclinical studies in a disease model are scarce. Recently, Kinross et al. systemically overexpressed the H1047R mutation of PIK3CA, leading to increased body weight with enlarged organs due to increased cell number [53]. In addition, Hare et al. overexpressed the PIK3CA H1047R mutation specifically in endothelial cells, leading to extensive vascular remodeling and embryonic lethality [54]. 

## 5. PI3Ks as a Master Controller of Proliferation in the Kidney and in Polycystic Kidney Disease

Besides cancer [1] and overgrowth syndromes [44], alterations in the PI3K/AKT/mTOR pathway are major drivers of abnormal proliferation. In the kidney, the hyper-proliferation of tubular cells induces renal cyst formation, which can be considered a sort of overgrowth syndrome and is typical of polycystic kidney disease (PKD). In this context, the increased proliferation can be caused by opposite genetic lesions occurring in PI3Ks: on one side, hyper-activation of the AKT-dependent class I PI3KCA signaling pathway, on the other side, loss of class II PI3K-C2α. 

In PKD, it is well described that alterations of several components of the PI3K/AKT/mTOR pathway, already found in cancer, are implicated in the hyper-proliferation of renal tubular cells, such as TSC. TSC is a well-known tumor suppressor, which is negatively regulated by AKT and acts as a GTPase Activating Protein (GAP) for the small GTPase Rheb, thus leading to mTORC1 inhibition. TSC1 and TSC2 genes are mutated in a genetic disorder called tuberous sclerosis complex (TSC). TSC causes the development of hamartomas, which can arise in multiple organs, including brain, kidney, skin and liver. Humans and mice with loss of either TSC1 or TSC2 are prone to developing renal problems including cysts, which usually do not interfere with kidney function [55]. However, in a small subset of patients with loss of TSC and who were affected by severe infantile polycystic kidney disease, cysts led to renal failure [56]. It was reported that conditional knockout of TSC1 in renal tubular cells resulted in PKD [57]. Both the hyper-activation of class I PI3KCA and the inhibition of TSC result in the mTOR-mediated increase in cell proliferation. Besides its AKT-mediated inhibition, TSC can be activated by polycystins. Polycistin-1 acts as a mechanosensor of the fluid flux and activates the calcium channel Polycistin-2. These two transmembrane proteins are encoded by PKD1 or PKD2 genes, which are mutated in autosomal-dominant polycystic kidney disease (ADPKD), a genetic disorder characterized by uncontrolled proliferation of renal cells with the consequent formation of cysts and loss of renal function [58,59]. The anti-proliferative signal of polycystins due to mTORC1 inhibition is mediated by TSC1 and TSC2 genes. Mice with concomitant loss of TSC and PC1 revealed that cyst development is closely correlated with elevated mTORC1 activity, leading to hypertrophy and increased proliferation of the renal tubular cells [60]. In cells, the coexistence of mutation and loss of TSC alleles presented an alteration in the localization of PC1 on the plasma membrane [57]. Re-expression of PC1 in the TSC1-mutant kidneys corrects the phenotype. On the other hand, mTORC1 acts in a negative feedback loop to downregulate PC1 expression levels. Consistently, the inhibition of mTOR increases the expression levels of polycystins and slows cyst expansion [60,61].

Conversely, mTOR activation is also triggered by the loss of a class II PI3K-C2α, which is important for the correct transport of Polycystins along the primary cilium. Polycystins exert their function only when properly localized on this organelle [62]. The localization of PC2, in turn, is required for a correct transduction of the anti-proliferative signal, inhibiting intracellular pathways, including the mTOR and mitogen-activated protein kinase (MAPK) pathways, eventually avoiding cystogenesis [63]. The transport of PC2 along the primary cilium is mediated by the small guanosine triphosphatase (GTPase) RAB8, which in turn is regulated by RAB11. It was demonstrated that PI3K-C2α acts at the pericentriolar recycling endosomal compartment (PRE) at the base of the primary cilium where it produces PI(3)P and promotes RAB11 activation upstream of RAB8 [6,7,64]. The inhibition of PI3K-C2α decreases the number of PC2-positive cilia, resulting in increased mTOR and MAPK signaling. Accordingly, the expression of a constitutive active form of RAB8 (RAB8^Q67L^) is able to restore these defects. Indeed, the concomitant heterozygous loss of PI3K-C2α and PC1 or PC2 correlates with a higher number of cysts in proximal and distant tubules. These data indicate that PI3K-C2α activates the RAB11/RAB8 cascade, regulating the transport of cargo proteins, such as polycystins, along the primary cilium, thus preventing cyst formation [7]. These data point out that uncontrolled proliferation is due to the hyper-activation of class I PI3KCA or loss of class II PI3K-C2α. For class I PI3K, the proliferation signal is induced by the PI(3,4,5)P3-mediated activation of AKT, which in turn enhances mTOR activity. On the contrary, for class II PI3K, cell proliferation seems to be triggered by polycystin-mediated inhibition of mTOR. The apparent contradiction of these different PI3Ks could be explained by the lack of the AKT-activating PI(3,4,5)P3 production by class II PI3K. Consistently, PI3K-C2β knockout enhances insulin/AKT signaling in metabolic tissues, probably due to a compensatory enhancement of class I PI3K activation, which produces PI(3,4,5)P3 and activates AKT [65].

In the prevention of cyst formation, negative regulators of PI3Ks, including PTEN, are implicated. PTEN loss per se is not sufficient to induce cyst development [66]; conversely, the concomitant loss of PTEN and other tumor suppressor genes, such as Von Hippel-Lindau (VHL) and TSC, was implicated in cyst formation. VHL syndrome is a familiar cancer which predisposes to a variety of malignant and benign tumors. In some patients, kidney cysts are precursor lesions for clear cell renal cell carcinoma (ccRCC), one the most common malignant kidney tumors. Cysts in human VHL patients, but not VHL mutant cells, display hyper-activation of the PI3K/AKT/mTOR signaling pathways, indicating that PI3K mutations may be an early event in the transformation process. Double-knockout mice for PTEN and VHL showed enlarged and heavier kidneys due to multiple cysts, characterized by a lack of primary cilium, increased proliferation and activated PI3K signaling [67,68]. Similarly, it was reported that mice lacking PTEN and TSC1 develop severe PKD [66]. This suggests that concomitant mutations are needed to induce cyst development in a cancer-prone background.

A recent study identified a four-gene signature as a prognostic value for ccRCC, including PTEN, PIK3C2A, ITPA and BCL3 [69]. Moreover, low expression of PTEN and PIK3C2A was correlated with high risk and poor prognosis [69].

By now, the only available therapies for ADPKD are supportive treatments, such as regulation of blood pressure, and dialysis or kidney transplantation are often required. Given the concomitant alterations of pathways other than mTOR, the efficiency of molecular target therapies, such as sorafenib and temsirolimus, is limited to 10%–40%. Preclinical trials based on mTOR inhibition (mTOR-I) by either the immunosuppressant sirolimus (SIR) [61,70] or everolimus (EVER) [71] demonstrated that the sirolimus injection led to increased kidney size, cyst density and tubular cell proliferation. However, in clinical trials only negligible effects on cyst growth and preservation of renal function were observed, due to the development of resistance [72,73]. In particular, it was demonstrated that sirolimus treatment may restore a cell’s ability to activate AKT, suggesting that patients with TSC show a high risk of malignant tumor development due to long-term treatment [74]. Up to now, the most important supportive treatment for ADPKD is the regulation of blood pressure. A clinical trial for tolvaptan, a vasopressin 2 receptor (V2R) antagonist, is still under Food and Drug Administration (FDA) approval, due to the request for further information about side effects and selection of patient cohorts who may benefit from treatment (clinicaltrials.gov). As V2R is a GPCR-coupled receptor whose downstream pathway is mediated by PI3Ks, we can speculate that the targeting of PI3K itself, but not mTOR, could be beneficial in the treatment of ADPKD. 

## 6. Conclusions

PI3K/AKT/mTOR is frequently deregulated in several proliferative disorders, from cancer to overgrowth syndromes and polycystic kidney disease. Despite similar mutations, patients develop a plethora of different disorders. For example, PIK3CA mutations associated with overgrowth syndromes are often similar to those observed in cancer, but these patients do not seem to be predisposed to cancer. Similarly, loss of tumor suppressor genes such as TSC1 and TSC2 is not always accompanied by the development of renal cysts and overgrowth. This suggests that the role of mutations in disease is determined by the context in which they occur. Clinical variability may depend on the timing of the mutation during embryogenesis, the type of tissue affected and concomitant secondary lesions. On the other hand, the common molecular basis of these disorders is associated with significant clinical overlap. This will eventually help to apply the therapeutic strategies used for well-studied pathologies, such as cancer, to emerging clinically relevant diseases, such as overgrowth syndrome and polycystic kidney disease. Conversely, patients with ADPKD did not benefit from rapalogs treatment, indicating that mTOR-mediated signaling is not the only pathway involved in cyst formation. These clinical failures imply that a novel therapeutic option could be based on the single or combined targeting of PI3K pathway components.

## Figures and Tables

**Table 1:** PIK3CA-related overgrowth spectrum (PROS). PIK3CA predicted mutations and associated clinical overgrowth disorders (for a comprehensive study please see Keppler-Noreuil 2015) [31]).

Clinical PROS Disorder	Mutation (Gene Domain)	References
Megalencephaly-capillary malformation syndrome (MCAP)	E81K, R88Q (p85 BD) G364R, E365K, C378Y, E452K, E453K, E453del (C2) E542K, E545K (Helical) E726K G914R, Y1021C, T1025A, A1035V (Kinase) H1047L, H1047R, M1043I, H1047Y, G1049S (Kinase)	[32]
Congenital lipomatous overgrowth, vascular malformations, linear keratinocytic epidermal nevi, and skeletal or spinal anomalies (CLOVES) syndrome	C420R E542K, E545K (Helical) H1047L, H1047R (Kinase)	[24,37,38]
Fibroadipose overgrowth (FAO)/Hemihyperplasia multiple lipomatosis (HHML)	E542K, E545K (Helical) H1047L, H1047R (Kinase)	[39,40]
Macrodactyly	R115P E542K, E545K (Helical) H1047L, H1047R (Kinase)	[41]
Hemimegalencephaly	E542K, E545K (Helical)	[42]
Muscle hemihyperplasia	E542K, E545K (Helical) H1047L, H1047R, L1067fs (Kinase)	[41]
Facial infiltrating lipomatosis	E452K, E453K, E453del (C2) E542K, E545K (Helical) H1047L, H1047R (Kinase)	[43]
Epidermal nevi	E542K, E545K (Helical) H1047L, H1047R (Kinase)	[19]
Seborrheic keratoses	E542K, E545K (Helical) H1047L, H1047R (Kinase)	[19]
Benign lichenoid keratoses	E542K, E545K (Helical)	[19]

## References

[1] Martini M., De Santis M.C., Braccini L., Gulluni F., Hirsch E. (2014). PI3K/AKT signaling pathway and cancer: An updated review. Ann. Med..

[2] Vanhaesebroeck B., Guillermet-Guibert J., Graupera M., Bilanges B. (2010). The emerging mechanisms of isoform-specific PI3K signalling. Nat. Rev. Mol. Cell Biol..

[3] Falasca M., Maffucci T. (2012). Regulation and cellular functions of class II phosphoinositide 3-kinases. Biochem. J..

[4] Campa C.C., Martini M., De Santis M.C., Hirsch E. (2015). How PI3K-derived lipids control cell division. Front. Cell Dev. Biol..

[5] Shibazaki S., Yu Z., Nishio S., Tian X., Thomson R.B., Mitobe M., Louvi A., Velazquez H., Ishibe S., Cantley L.G. (2008). Cyst formation and activation of the extracellular regulated kinase pathway after kidney specific inactivation of pkd1. Hum. Mol. Genet..

[6] Franco I., Gulluni F., Campa C.C., Costa C., Margaria J.P., Ciraolo E., Martini M., Monteyne D., De Luca E., Germena G. (2014). PI3K class II alpha controls spatially restricted endosomal PtdIns3P and Rab11 activation to promote primary cilium function. Dev. Cell.

[7] Franco I., Margaria J.P., De Santis M.C., Ranghino A., Monteyne D., Chiaravalli M., Pema M., Campa C.C., Ratto E., Gulluni F. (2016). Phosphoinositide 3-Kinase-C2alpha regulates polycystin-2 ciliary entry and protects against kidney cyst formation. J. Am. Soc. Nephrol..

[8] Vivanco I., Sawyers C.L. (2002). The phosphatidylinositol 3-kinase AKT pathway in human cancer. Nat. Rev. Cancer.

[9] Weigelt B., Downward J. (2012). Genomic determinants of PI3K pathway inhibitor response in cancer. Front. Oncol..

[10] Samuels Y., Waldman T. (2010). Oncogenic mutations of PIK3CA in human cancers. Curr. Top. Microbiol. Immunol..

[11] Burke J.E., Perisic O., Masson G.R., Vadas O., Williams R.L. (2012). Oncogenic mutations mimic and enhance dynamic events in the natural activation of phosphoinositide 3-kinase p110alpha (PIK3CA). Proc. Natl. Acad. Sci. USA.

[12] Engelman J.A., Chen L., Tan X., Crosby K., Guimaraes A.R., Upadhyay R., Maira M., McNamara K., Perera S.A., Song Y. (2008). Effective use of PI3K and MEK inhibitors to treat mutant Kras G12D and PIK3CA H1047R murine lung cancers. Nat. Med..

[13] Gymnopoulos M., Elsliger M.A., Vogt P.K. (2007). Rare cancer-specific mutations in PIK3CA show gain of function. Proc. Natl. Acad. Sci. USA.

[14] Meyer D.S., Brinkhaus H., Muller U., Muller M., Cardiff R.D., Bentires-Alj M. (2011). Luminal expression of PIK3CA mutant H1047R in the mammary gland induces heterogeneous tumors. Cancer Res..

[15] Burke J.E., Williams R.L. (2013). Dynamic steps in receptor tyrosine kinase mediated activation of class ia phosphoinositide 3-kinases (PI3K) captured by H/D exchange (HDX-MS). Adv. Biol. Regul..

[16] Carson J.D., Van Aller G., Lehr R., Sinnamon R.H., Kirkpatrick R.B., Auger K.R., Dhanak D., Copeland R.A., Gontarek R.R., Tummino P.J. (2008). Effects of oncogenic p110alpha subunit mutations on the lipid kinase activity of phosphoinositide 3-kinase. Biochem. J..

[17] Samuels Y., Velculescu V.E. (2004). Oncogenic mutations of PIK3CA in human cancers. Cell Cycle.

[18] Oda K., Stokoe D., Taketani Y., McCormick F. (2005). High frequency of coexistent mutations of PIK3CA and PTEN genes in endometrial carcinoma. Cancer Res..

[19] Hafner C., Lopez-Knowles E., Luis N.M., Toll A., Baselga E., Fernandez-Casado A., Hernandez S., Ribe A., Mentzel T., Stoehr R. (2007). Oncogenic PIK3CA mutations occur in epidermal nevi and seborrheic keratoses with a characteristic mutation pattern. Proc. Natl. Acad. Sci. USA.

[20] Hafner C., Stoehr R., van Oers J.M., Zwarthoff E.C., Hofstaedter F., Klein C., Landthaler M., Hartmann A., Vogt T. (2009). The absence of BRAF, FGFR3, and PIK3CA mutations differentiates lentigo simplex from melanocytic nevus and solar lentigo. J. Investig. Dermatol..

[21] Omholt K., Krockel D., Ringborg U., Hansson J. (2006). Mutations of PIK3CA are rare in cutaneous melanoma. Melanoma Res..

[22] Van Steensel M.A. (2015). Neurocutaneous manifestations of genetic mosaicism. J. Pediatr. Genet..

[23] Bhagirath D., Zhao X., West W.W., Qiu F., Band H., Band V. (2015). Cell type of origin as well as genetic alterations contribute to breast cancer phenotypes. Oncotarget.

[24] Kurek K.C., Luks V.L., Ayturk U.M., Alomari A.I., Fishman S.J., Spencer S.A., Mulliken J.B., Bowen M.E., Yamamoto G.L., Kozakewich H.P. (2012). Somatic mosaic activating mutations in PIK3CA cause cloves syndrome. Am. J. Hum. Genet..

[25] Keppler-Noreuil K.M., Sapp J.C., Lindhurst M.J., Parker V.E., Blumhorst C., Darling T., Tosi L.L., Huson S.M., Whitehouse R.W., Jakkula E. (2014). Clinical delineation and natural history of the PIK3CA-related overgrowth spectrum. Am. J. Med. Genet. A.

[26] Acuna-Hidalgo R., Veltman J.A., Hoischen A. (2016). New insights into the generation and role of de novo mutations in health and disease. Genome Biol..

[27] Aoki Y., Niihori T., Inoue S., Matsubara Y. (2016). Recent advances in rasopathies. J. Hum. Genet..

[28] Hafner C., Groesser L. (2013). Mosaic rasopathies. Cell Cycle.

[29] Edmondson A.C., Kalish J.M. (2015). Overgrowth syndromes. J. Pediatr. Genet..

[30] Loconte D.C., Grossi V., Bozzao C., Forte G., Bagnulo R., Stella A., Lastella P., Cutrone M., Benedicenti F., Susca F.C. (2015). Molecular and functional characterization of three different postzygotic mutations in PIK3CA-related overgrowth spectrum (PROS) patients: Effects on Pi3k/Akt/mTOR signaling and sensitivity to PIK3 inhibitors. PLoS ONE.

[31] Keppler-Noreuil K.M., Rios J.J., Parker V.E., Semple R.K., Lindhurst M.J., Sapp J.C., Alomari A., Ezaki M., Dobyns W., Biesecker L.G. (2015). PIK3CA-related overgrowth spectrum (PROS): Diagnostic and testing eligibility criteria, differential diagnosis, and evaluation. Am. J. Med. Genet. A.

[32] Riviere J.B., Mirzaa G.M., O’Roak B.J., Beddaoui M., Alcantara D., Conway R.L., St-Onge J., Schwartzentruber J.A., Gripp K.W., Nikkel S.M. (2012). De novo germline and postzygotic mutations in AKT3, PIK3R2 and PIK3CA cause a spectrum of related megalencephaly syndromes. Nat. Genet..

[33] Bubien V., Bonnet F., Brouste V., Hoppe S., Barouk-Simonet E., David A., Edery P., Bottani A., Layet V., Caron O. (2013). High cumulative risks of cancer in patients with PTEN hamartoma tumour syndrome. J. Med. Genet..

[34] Caux F., Plauchu H., Chibon F., Faivre L., Fain O., Vabres P., Bonnet F., Selma Z.B., Laroche L., Gerard M. (2007). Segmental overgrowth, lipomatosis, arteriovenous malformation and epidermal nevus (solamen) syndrome is related to mosaic PTEN nullizygosity. Eur. J. Hum. Genet..

[35] Chimowitz M.I., Lynn M.J., Derdeyn C.P., Turan T.N., Fiorella D., Lane B.F., Janis L.S., Lutsep H.L., Barnwell S.L., Waters M.F. (2011). Stenting versus aggressive medical therapy for intracranial arterial stenosis. N. Engl. J. Med..

[36] Hussain K., Challis B., Rocha N., Payne F., Minic M., Thompson A., Daly A., Scott C., Harris J., Smillie B.J. (2011). An activating mutation of AKT2 and human hypoglycemia. Science.

[37] Sapp J.C., Turner J.T., van de Kamp J.M., van Dijk F.S., Lowry R.B., Biesecker L.G. (2007). Newly delineated syndrome of congenital lipomatous overgrowth, vascular malformations, and epidermal nevi (CLOVE syndrome) in seven patients. Am. J. Med. Genet. A.

[38] Alomari A.I. (2009). Characterization of a distinct syndrome that associates complex truncal overgrowth, vascular, and acral anomalies: A descriptive study of 18 cases of cloves syndrome. Clin. Dysmorphol..

[39] Lindhurst M.J., Parker V.E., Payne F., Sapp J.C., Rudge S., Harris J., Witkowski A.M., Zhang Q., Groeneveld M.P., Scott C.E. (2012). Mosaic overgrowth with fibroadipose hyperplasia is caused by somatic activating mutations in PIK3CA. Nat. Genet..

[40] Biesecker L.G., Peters K.F., Darling T.N., Choyke P., Hill S., Schimke N., Cunningham M., Meltzer P., Cohen M.M. (1998). Clinical differentiation between proteus syndrome and hemihyperplasia: Description of a distinct form of hemihyperplasia. Am. J. Med. Genet..

[41] Rios J.J., Paria N., Burns D.K., Israel B.A., Cornelia R., Wise C.A., Ezaki M. (2013). Somatic gain-of-function mutations in PIK3CA in patients with macrodactyly. Hum. Mol. Genet..

[42] Lee J.H., Huynh M., Silhavy J.L., Kim S., Dixon-Salazar T., Heiberg A., Scott E., Bafna V., Hill K.J., Collazo A. (2012). De novo somatic mutations in components of the PI3K-AKT3-mTOR pathway cause hemimegalencephaly. Nat. Genet..

[43] Maclellan R.A., Luks V.L., Vivero M.P., Mulliken J.B., Zurakowski D., Padwa B.L., Warman M.L., Greene A.K., Kurek K.C. (2014). Pik3ca activating mutations in facial infiltrating lipomatosis. Plast. Reconstr. Surg..

[44] Keppler-Noreuil K.M., Parker V.E., Darling T.N., Martinez-Agosto J.A. (2016). Somatic overgrowth disorders of the PI3K/AKT/mTOR pathway & therapeutic strategies. Am. J. Med. Genet. C Semin. Med. Genet..

[45] Castel P., Carmona F.J., Grego-Bessa J., Berger M.F., Viale A., Anderson K.V., Bague S., Scaltriti M., Antonescu C.R., Baselga E. (2016). Somatic PIK3CA mutations as a driver of sporadic venous malformations. Sci. Transl. Med..

[46] Castillo S.D., Tzouanacou E., Zaw-Thin M., Berenjeno I.M., Parker V.E., Chivite I., Mila-Guasch M., Pearce W., Solomon I., Angulo-Urarte A. (2016). Somatic activating mutations in PIK3CA cause sporadic venous malformations in mice and humans. Sci. Transl. Med..

[47] Lamming D.W., Ye L., Sabatini D.M., Baur J.A. (2013). Rapalogs and mTOR inhibitors as anti-aging therapeutics. J. Clin. Investig..

[48] Boon L.M., Hammer J., Seront E., Dupont S., Hammer F., Clapuyt P., Vikkula M. (2015). Rapamycin as novel treatment for refractory-to-standard-care slow-flow vascular malformations. Plast. Reconstr. Surg..

[49] Lackner H., Karastaneva A., Schwinger W., Benesch M., Sovinz P., Seidel M., Sperl D., Lanz S., Haxhija E., Reiterer F. (2015). Sirolimus for the treatment of children with various complicated vascular anomalies. Eur. J. Pediatr..

[50] Marsh D.J., Trahair T.N., Martin J.L., Chee W.Y., Walker J., Kirk E.P., Baxter R.C., Marshall G.M. (2008). Rapamycin treatment for a child with germline PTEN mutation. Nat. Clin. Pract. Oncol..

[51] Schmid G.L., Kassner F., Uhlig H.H., Korner A., Kratzsch J., Handel N., Zepp F.P., Kowalzik F., Laner A., Starke S. (2014). Sirolimus treatment of severe PTEN hamartoma tumor syndrome: Case report and in vitro studies. Pediatr. Res..

[52] Maira S.M., Stauffer F., Brueggen J., Furet P., Schnell C., Fritsch C., Brachmann S., Chene P., De Pover A., Schoemaker K. (2008). Identification and characterization of NVP-BEZ235, a new orally available dual phosphatidylinositol 3-kinase/mammalian target of rapamycin inhibitor with potent in vivo antitumor activity. Mol. Cancer Ther..

[53] Kinross K.M., Montgomery K.G., Mangiafico S.P., Hare L.M., Kleinschmidt M., Bywater M.J., Poulton I.J., Vrahnas C., Henneicke H., Malaterre J. (2015). Ubiquitous expression of the Pik3caH1047R mutation promotes hypoglycemia, hypoinsulinemia, and organomegaly. FASEB J..

[54] Hare L.M., Schwarz Q., Wiszniak S., Gurung R., Montgomery K.G., Mitchell C.A., Phillips W.A. (2015). Heterozygous expression of the oncogenic Pik3ca(H1047R) mutation during murine development results in fatal embryonic and extraembryonic defects. Dev. Biol..

[55] Crino P.B., Nathanson K.L., Henske E.P. (2006). The tuberous sclerosis complex. N. Engl. J. Med..

[56] Traykova-Brauch M., Schonig K., Greiner O., Miloud T., Jauch A., Bode M., Felsher D.W., Glick A.B., Kwiatkowski D.J., Bujard H. (2008). An efficient and versatile system for acute and chronic modulation of renal tubular function in transgenic mice. Nat. Med..

[57] Armour E.A., Carson R.P., Ess K.C. (2012). Cystogenesis and elongated primary cilia in Tsc1-deficient distal convoluted tubules. Am. J. Physiol. Renal. Physiol..

[58] Harris P.C., Torres V.E. (2009). Polycystic kidney disease. Annu. Rev. Med..

[59] Igarashi P., Somlo S. (2002). Genetics and pathogenesis of polycystic kidney disease. J. Am. Soc. Nephrol..

[60] Pema M., Drusian L., Chiaravalli M., Castelli M., Yao Q., Ricciardi S., Somlo S., Qian F., Biffo S., Boletta A. (2016). mTORC1-mediated inhibition of polycystin-1 expression drives renal cyst formation in tuberous sclerosis complex. Nat. Commun..

[61] Tao Y., Kim J., Schrier R.W., Edelstein C.L. (2005). Rapamycin markedly slows disease progression in a rat model of polycystic kidney disease. J. Am. Soc. Nephrol..

[62] Hildebrandt F., Benzing T., Katsanis N. (2011). Ciliopathies. N. Engl. J. Med..

[63] Ma M., Tian X., Igarashi P., Pazour G.J., Somlo S. (2013). Loss of cilia suppresses cyst growth in genetic models of autosomal dominant polycystic kidney disease. Nat. Genet..

[64] Campa C.C., Franco I., Hirsch E. (2015). PI3K-C2alpha: One enzyme for two products coupling vesicle trafficking and signal transduction. FEBS Lett..

[65] Alliouachene S., Bilanges B., Chicanne G., Anderson K.E., Pearce W., Ali K., Valet C., Posor Y., Low P.C., Chaussade C. (2015). Inactivation of the class II PI3K-C2beta potentiates insulin signaling and sensitivity. Cell Rep..

[66] Zhou J., Brugarolas J., Parada L.F. (2009). Loss of Tsc1, but not pten, in renal tubular cells causes polycystic kidney disease by activating mTORC1. Hum. Mol. Genet..

[67] Thoma C.R., Frew I.J., Hoerner C.R., Montani M., Moch H., Krek W. (2007). pVHL and GSK3beta are components of a primary cilium-maintenance signalling network. Nat. Cell Biol..

[68] Frew I.J., Thoma C.R., Georgiev S., Minola A., Hitz M., Montani M., Moch H., Krek W. (2008). pVHL and PTEN tumour suppressor proteins cooperatively suppress kidney cyst formation. EMBO J..

[69] Dai J., Lu Y., Wang J., Yang L., Han Y., Wang Y., Yan D., Ruan Q., Wang S. (2016). A four-gene signature predicts survival in clear-cell renal-cell carcinoma. Oncotarget.

[70] Serra A.L., Poster D., Kistler A.D., Krauer F., Raina S., Young J., Rentsch K.M., Spanaus K.S., Senn O., Kristanto P. (2010). Sirolimus and kidney growth in autosomal dominant polycystic kidney disease. N. Engl. J. Med..

[71] Walz G., Budde K., Mannaa M., Nurnberger J., Wanner C., Sommerer C., Kunzendorf U., Banas B., Horl W.H., Obermuller N. (2010). Everolimus in patients with autosomal dominant polycystic kidney disease. N. Engl. J. Med..

[72] Torres V.E., Boletta A., Chapman A., Gattone V., Pei Y., Qian Q., Wallace D.P., Weimbs T., Wuthrich R.P. (2010). Prospects for mTOR inhibitor use in patients with polycystic kidney disease and hamartomatous diseases. Clin. J. Am. Soc. Nephrol..

[73] Lieberthal W., Fuhro R., Andry C., Patel V., Levine J.S. (2006). Rapamycin delays but does not prevent recovery from acute renal failure: Role of acquired tubular resistance. Transplantation.

[74] Mathewson H.O. (1967). Two experimental general practices. United KIngdom and United States. Arch. Environ. Health.

